# Inflammasome and Its Therapeutic Targeting in Rheumatoid Arthritis

**DOI:** 10.3389/fimmu.2021.816839

**Published:** 2022-01-13

**Authors:** Qi Jiang, Xin Wang, Enyu Huang, Qiao Wang, Chengping Wen, Guocan Yang, Liwei Lu, Dawei Cui

**Affiliations:** ^1^ Department of Blood Transfusion, Shaoxing People’s Hospital (Shaoxing Hospital, Zhejiang University School of Medicine), Shaoxing, China; ^2^ Department of Rheumatology and Immunology, Shaoxing People’s Hospital (Shaoxing Hospital, Zhejiang University School of Medicine), Shaoxing, China; ^3^ Department of Pathology and Shenzhen Institute of Research and Innovation, The University of Hong Kong, Hong Kong, Hong Kong SAR, China; ^4^ Chongqing International Institute for Immunology, Chongqing, China; ^5^ School of Basic Medical Science, Zhejiang Chinese Medical University, Hangzhou, China; ^6^ Department of Blood Transfusion, The First Affiliated Hospital, Zhejiang University School of Medicine, Hangzhou, China

**Keywords:** inflammasome, autoimmunity, immunotherapy, rheumatoid arthritis, inflammation

## Abstract

Inflammasome is a cytoplasmic multiprotein complex that facilitates the clearance of exogenous microorganisms or the recognition of endogenous danger signals, which is critically involved in innate inflammatory response. Excessive or abnormal activation of inflammasomes has been shown to contribute to the development of various diseases including autoimmune diseases, neurodegenerative changes, and cancers. Rheumatoid arthritis (RA) is a chronic and complex autoimmune disease, in which inflammasome activation plays a pivotal role in immune dysregulation and joint inflammation. This review summarizes recent findings on inflammasome activation and its effector mechanisms in the pathogenesis of RA and potential development of therapeutic targeting of inflammasome for the immunotherapy of RA.

## Introduction

Rheumatoid arthritis (RA) is a chronic and systemic autoimmune disease that manifests as persistent inflammation of the synovial joints, leading to synovial tissue proliferation, cartilage erosion and consequent joint deformation with functional limitations (1–3). RA occurs at any age, there are more than 20 million prevalent cases of RA, given the general increase in life expectancy worldwide, and the number of elderly patients with RA is increasing annually. Globally, the age-standardized point annual incidence of RA has increased by 8.2% compared to 1990 (4, 5). The ratio of male patients to female patients with RA is approximately 1:3, which is possibly associated with the stimulation of the immune system by estrogen (6, 7). The onset of RA is also associated with pregnancy and menopause (8, 9). Although the exact pathogenesis of RA remains unclear, genetics, smoking, obesity, infections, periodontal disease, and even gut microbiota are currently thought to be associated with the development of RA (10, 11). For example, the HLA-DRB1 gene within the human leukocyte antigen (HLA) locus is associated with increased susceptibility and severity of RA, although genetic susceptibility factors for RA are significantly different between Asian and European populations, the HLA-DRB1 gene is a common susceptibility gene in all populations (12, 13). The DRB1 shared epitope allele also synergizes with smoking and increases the risk of anti-citrullinated protein antibody (ACPA)-positive RA (14). The levels of anti-cyclic citrullinated peptide (CCP) IgG antibodies, rheumatoid factor (RF), erythrocyte sedimentation rate (ESR) and C-reactive protein (CRP) in blood reflect the extent of inflammation and tissue damage in RA patients (15). Interleukin (IL)-1β, IL-6, IL-18 and tumor necrosis factor (TNF) are the major proinflammatory cytokines in RA. IL-1β enhances the secretion of chemokines and cytokines, promotes Th17 cell differentiation and reduces the synthesis of cartilage components (15). Moreover, the severity of RA is positively correlated with serum IL-18 levels (16–18). These findings indicate an important role of inflammation in the pathogenesis of RA.

The human immune system is composed of the innate immune system and the adaptive immune system. The innate immune system consists of anatomical barriers (skin, mucous membranes), hematopoietic cells (such as macrophages, dendritic cells, monocytes), nonhematopoietic cells (such as epithelial cells), and the complement system (19). Unlike the adaptive immune cells, which are antigen-specific and capable of generating immunological memory, innate immune cells are pre-programmed to recognize molecules shared by broad categories of pathogens or pathological situations, such as pathogen-associated molecular patterns (PAMPs), damage-associated molecular patterns (DAMPs), homeostasis-altering molecular processes (HAMPs), and pattern recognition receptors (PRRs) (20, 21). Based on their locations, PRRs are categorized into membrane PRRs, cytoplasmic PRRs, and secretory PRRs. Based on their structures, PRRs are categorized into Toll-like receptors (TLRs), NOD-like receptors (NLRs), c-type lectin receptors (CLRs), and retinoic acid-inducible gene (RIG)-I-like receptors (RLRs) (22, 23). NLRs are cytoplasmic PRRs that play a bridging role between innate and adaptive immunity by activating a variety of inflammatory factor precursors and inducing the release of inflammatory factors (22, 23). Inflammation itself is a protective mechanism of the organism in response to internal and external stimuli; a moderate inflammatory response contributes to the stability of the body’s internal environment, whereas excessive or persistent inflammation will lead to cancer or other diseases (22, 23). Since the early response to inflammatory reactions is achieved mainly by stimulating inflammasomes, it is particularly important to understand the activation process of inflammasomes (24–26). However, current knowledge of about the role of inflammasomes in the pathogenesis of RA remains incomplete. This review will systematically describe the classification, structure, and activation mechanisms of inflammasomes and discuss about the role of inflammasomes and their therapeutic targeting in RA treatment.

## Overview of Inflammasomes

**Figure 1:**
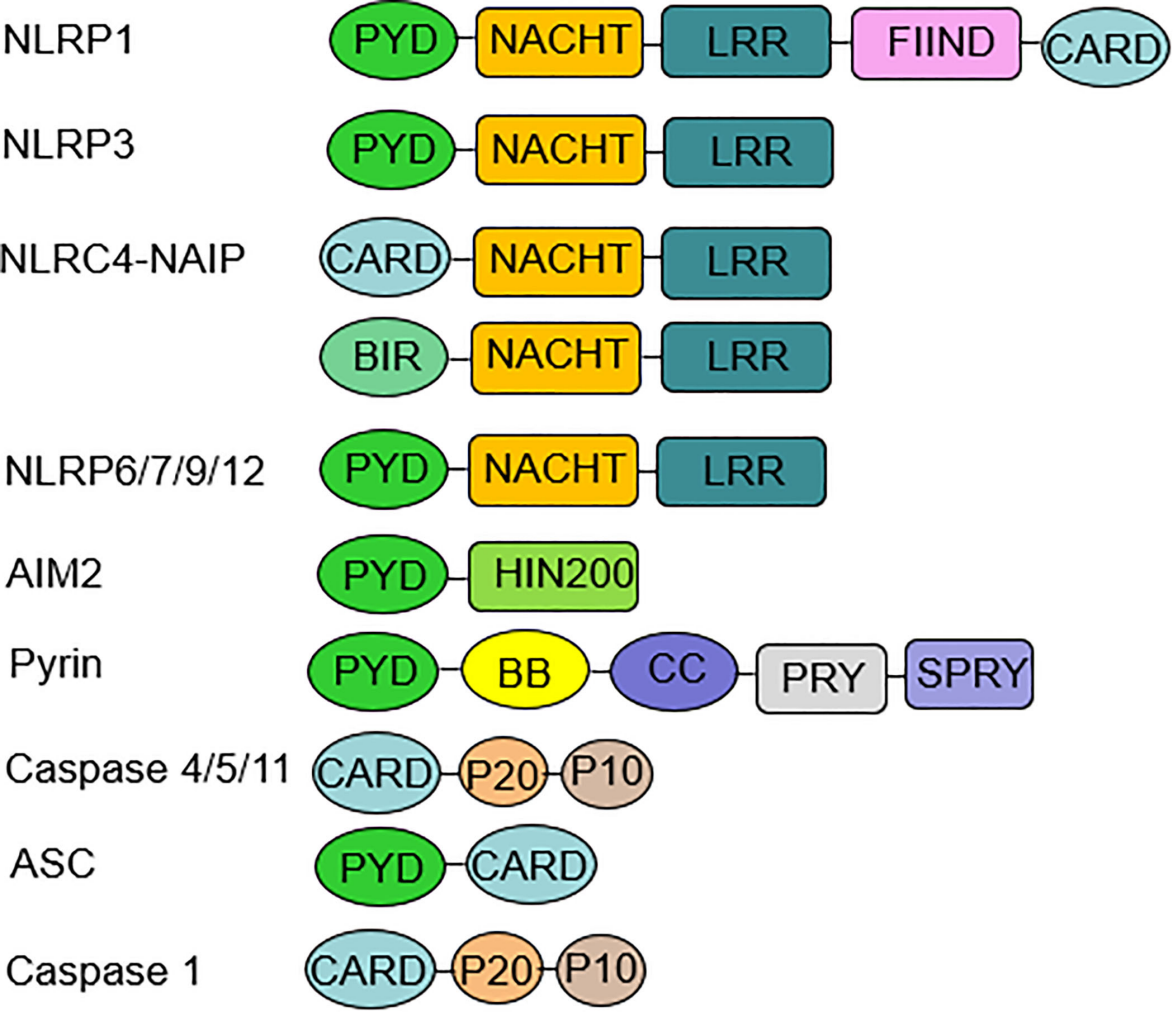
Inflammasome components and domain structure. Inflammasome complexes are formed by the oligomerization of several protein domains. A typical inflammasome consists of three parts: a sensor molecule, the adaptor protein ASC and the effector molecule pro-caspase-1. The sensors include NLRs, AIM2, and pyrin. NLRP3, 6, 7, 9, and 12 sensors all have a PYD at the N-terminus, an NBD or NACHT in the middle, and an LRR domain at the C-terminus. AIM2 consists of a PYD at the N-terminus and a HIN-200 domain at the C-terminus. ASC is required for the formation of NLRP3, 6, 7, 9, 12, and AIM2 inflammasomes. ASC mediates signaling to promote pro-caspase-1 activation through homotypic PYD-PYD or CARD-CARD interactions. The NLRP1 sensor also has a PYD at the N-terminus and a CARD at the C-terminus, which can bind directly to caspase-1 independent of ASC, and NLRP1 has a unique FIIND domain that is involved in inflammasome activation through its own protein hydrolysis. NLRC4 has an N-terminal CARD, an intermediate NBD or NACHT, and a C-terminal LRR domain. NAIP is required for NLRC4 to recognize PAMP, and then NLRC4 self-activates and oligomerizes to form the NAIP-NLRC4 inflammasome. Noncanonical inflammasomes include human caspase-4, caspase-5, and murine caspase-11. *ASC, apoptosis-associated speck-like protein containing a caspase recruitment domain; AIM2, absent in melanoma AIM2; PYD, pyrin domain; NBD, nucleotide-binding domain; CARD, caspase recruitment domain; PAMP, pathogen-associated molecular patterns*.

**Figure 2:**
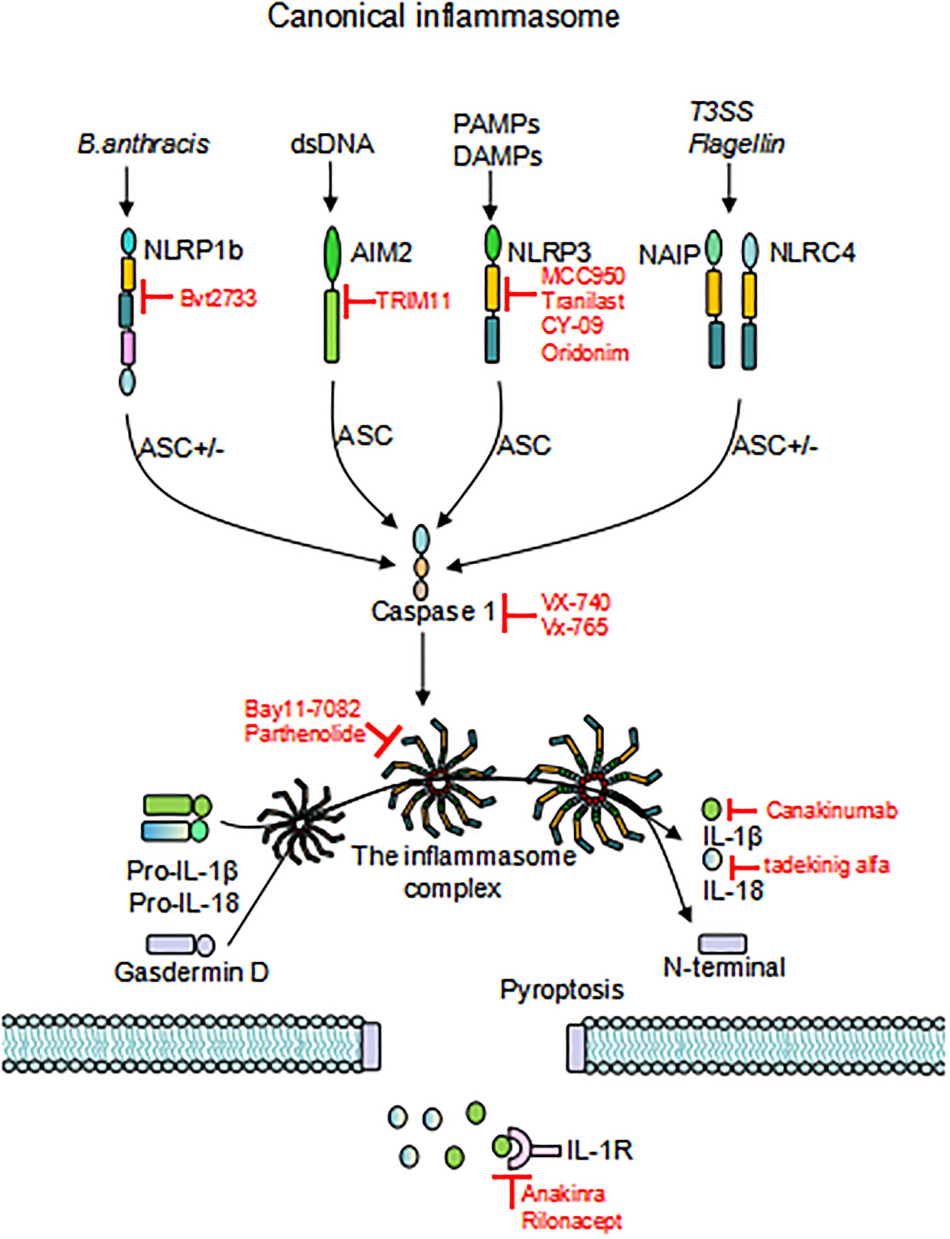
Activation of inflammasomes. Different inflammasome sensors sense different ligands, canonical inflammasome activation occurs through the response to PAMP/DAMP/HAMP. Upon initiation and activation, the inflammasome complex assembles, inducing caspase-1 self-cleavage and activation, and cleaves GSDMD to release the N-terminal domain and induce pyroptosis. Activated caspase-1 promotes cleavage of pro-IL-1β and pro-IL-18 to activate IL-1β and IL-18, which are released through the GSDMD pore. Inhibitors that target inflammasomes are also depicted in the Figure. * “ASC+/-”means this activation pathway is independent of ASC; PAMP, pathogen-associated molecular patterns; DAMP, damage-associated molecular patterns; HAMP, homeostasis-altering molecular processes; GSDMD, gasdermin D*.

In 2002, Martinon et al. firstly described inflammasomes as multiprotein platforms formed by organisms in response to various pathogenic or physiological factors (27). These oligomeric protein complexes can respond to a variety of ligands and have unique activation and regulatory mechanisms. There are two types of inflammasomes: the canonical inflammasomes that activate caspase-1, including NLRP1, NLRP3, NOD-like receptor family apoptosis inhibitory protein (NAIP)-NLRC4, NLRP6, NLRP7, NLRP9, NLRP12, absent in melanoma (AIM) 2, and pyrin inflammasomes; The noncanonical inflammasomes can activate caspase-4/5 (human) or caspase-11 (murine) (27–30) **(** **Figure 1** **)**. Canonical inflammasomes are composed of three components: a sensor molecule (responsible for DAMP/PAMP recognition), an adapter protein [apoptosis-associated speck-like protein containing a caspase recruitment domain (ASC)], and an effector molecule (pro-caspase-1) (31). The sensors include members of the NLR family, the AIM2-like receptor (ALR) family and pyrin, recognizing specific ligands to promote the assembly of inflammasomes. NLRs are composed of the N-terminal effector domains, pyrin domain (PYD) or caspase recruitment domain (CARD) or baculovirus inhibitor of apoptosis protein repeat (BIR), the central nucleotide-binding domain (NBD) or NACHT domain and the C-terminal leucine-rich repeat (LRR). According to the N-terminal domains, NLRs can be divided into five subfamilies: NLRA, NLRB, NLRC, NLRP and NLRX1. The human genome contains 23 NLR genes, and the mouse genome contains more than 30 NLR genes. These genes are expressed in a variety of tissues and cells; however, only a few NLR proteins form inflammasomes, and most known inflammasomes contain NLR structures (32–34). Sensors of the ALR family include AIM2 and human interferon (IFN)-g-inducible protein (IFI)16 (29, 35). ASC consists of a PYD and a CARD. ASCs mediate the oligomerization of components of inflammasomes and the signaling for caspase activation through homotypic PYD-PYD or CARD-CARD interactions coupled to upstream PRRs. These inflammasomes that require the adapter protein ASC for activation and assembly are called ASC-dependent inflammasomes, including NLRP3 and AIM2. ASC-independent inflammasomes such as NLRP1 and NAIP-NLRC4 directly activate caspase-1 *via* the CARD domain (36, 37). Caspases that are involved in inflammatory responses include human caspases-1, caspases-4 and caspases-5, as well as mouse caspases-1 and caspases-11 (38, 39). Active inflammatory caspases exert proinflammatory effects by cleaving pro-IL-1β and pro-IL-18 into active IL-1β and IL-18 *via* protein hydrolysis and promote pore-forming protein gasdermin D (GSDMD) cleavage to induce pyroptosis (38) **(** **Figure 2** **)**. This review focuses on the accumulation of PRR on the caspase-1 activation platform and inflammasomes in RA. This platform regulates the synthesis and activation of IL-1β and IL-18, which are the main inflammatory cytokines involved in RA.

### NLRP3

NLRP3 is an important inflammasomethathas beenextensively investigated and can be activated by multiple factors. Endogenous molecules include adenosine triphosphate (ATP), the heat shock protein HSP70, and uric acid crystals monosodium urate (MSU). Exogenous activators include lipopolysaccharides (LPS), components of the cell wall, microbial-specific nucleic acid structures, *Candida albicans*, and influenza viruses (40–44). Generally, NLRP3 has three distinct activation mechanisms, including altered ion flow (K^+^efflux, Cl^-^ flow and Ca^+^ flow), mitochondria-derived ROS production, and lysosomal rupture (45–49). Activation of typical NLRP3 requires two steps: microbial or endogenous cytokines bind to cell membrane receptors to activate nuclear factor-κB (NF-κB), increasing the expression levels of NLRP3 and pro-IL-1β, and then PAMPs/DAMPs/HAMPs trigger activation signals that recruit ASC and caspase-1 to form an inflammasome complex, which in turn drives caspase-1 self-cleavage and activation. Activated caspase-1 induces pro-IL-1β and pro-IL-18 cleavage to produce IL-1β and IL-18, which promote inflammatory responses, as well as shear GSDMD proteins, which induce pyrolysis (50, 51). Inflammasomes can also be activated *via* a noncanonical pathway. LPS release from gram-negative bacteria can lead to noncanonical inflammasome activation by initiating caspase-4/5 or murine caspase-11 signaling. Activation of these caspases promotes pore generation and K^+^ efflux from the cytoplasm to activate NLRP3 inflammasomes, which subsequently induce IL-1β and IL-18 maturation in a manner similar to the canonical inflammasome pathway (52–54). Recent studies have shown that Msn family kinase MINK1 is directly involved in regulating NLRP3 inflammasome (55).

### AIM2

AIM2 is well known for its ability to recognize intracellular double-stranded DNA (dsDNA), notably, host or pathogen-derived DNA in the cytoplasm (35). In normal cells, DNA is in the nucleus while the presence of DNA in the cytoplasm indicates compromised nuclear membrane integrity or infection (56). AIM2 is composed of two domains: the amino-terminal PYD and the carboxy-terminal HIN-200 domain (27). AIM2 binds to dsDNA in a sequence-independent manner, requiring a dsDNA length of 70 bp for activation in human and mouse cells, so the assembly of AIM2 is influenced by the length of the dsDNA (57, 58). Although AIM2 is considered as a cytoplasmic receptor, it is found that murine AIM2 is transported to the nucleus in response to ionizing radiation-induced DNA damage (59). However, in human monocytes, the cGAS-STING axis replaces the DNA inflammasome sensor function of AIM2 and triggers cell death in direct response to cytoplasmic DNA *via* the cGAS-STING-lysosome-NLRP3 pathway (60). In the presence of IFN-α, AIM2 can participate in the *Toxoplasma* response (61). All of these results suggest that AIM2 function in human cells may differ depending on the environment, and the exact mechanisms need to be further investigated. A recent study showed that AIM2 exacerbates atherosclerosis during clonal hematopoiesis and that treatment targeting inflammasomes may also reduce the risk of cardiovascular disease (62).

### NLRP1

Human NLRP1 was the first inflammasome to be identified and has two extra domains compared with NLRP3 **(** **Figure 1** **)**. The function-to-find (FIIND) domain is currently thought to be present in only two inflammasomes, NLRP1 and CARD8. The exact mechanism of the regulation of NLRP1 is unclear (63). Unlike human NLRP1, mouse NLRP1 carries three paralogs (a-c), of which NLRP1b has been most well characterized and can be activated by the anthrax lethal toxin produced by *Bacillus anthracis (* 64, 65). The eukaryote *Toxoplasma gondii* and the bacterium *Shigella flexneri* can activate human NLRP1 and murine NLRP1b (66, 67). Recent studies have shown that multiple viral proteases can also activate NLRP1 (68). Cytosolic dipeptidyl peptidase 9 (DPP9) can inhibit NLRP1 activation by closing the C-terminus of NLRP1, and the ZU5 domain is required for autoinhibition of human NLRP1 (69, 70). NLRP1 is the most prominent inflammasome manifested in human skin diseases. NLRP1 mutants lacking PYD are more likely to form ASC spots, whereas in AIM2 and NLRP3, PYD enhances ASC assembly, so the role of PYD varies in different inflammasomes (71).

### NAIP-NLRC4

The intermediate structure of NAIP-NLRC4 is NACHT (also known as NOD, a characteristic domain shared by the NLR family that mediates its oligomerization) with LRRs at the C-terminus that recognize and bind ligands (72). Human and mouse NAIP-NLRC4 can be activated by type three secretion system (T3SS) proteins and flagellin (73, 74). Only one NAIP protein isoform exists in humans, whereas mice have seven NAIP paralogs that can each bind different ligands (74). NAIP1 recognizes the T3SS needle protein, NAIP2 recognizes the T3SS inner rod protein, NAIP5 and NAIP6 recognize bacterial flagellin, and the ligands for NAIP3, 4, and 7 are currently unknown (75, 76). NAIP recognizes PAMP to activate NLRC4, and then NLRC4 self-activates, oligomerizes and forms NAIP-NLRC4 inflammasomes, causing a subsequent series of cascade reactions (77). Unlike NLRP3, NLRC4 activation is not ASC-dependent, and the secretion of IL-1β and IL-18 is reduced in the absence of ASC, while cell scorching is unaffected (78). These findings indicate that ASCs play a critical role in the secretion of proinflammatory cytokines.

### Other Inflammasomes

NLRP6 recruits ASC to form inflammasomes with caspase-1/11 and plays a role in intestinal diseases (79). The mRNA and protein levels of NLRP6 are reduced in fibroblast-like synovial cells (FLSs) and synovial tissue of RA patients. Overexpression of NLRP6 in RA-FLSs was associated with suppressed activation of NF-κB and reduced proinflammatory cytokines (80). NLRP6 is a negative regulator of inflammation in RA. There are no detailed reports on NLRP7, NLRP9, and pyrin inflammatory vesicles in RA. NLRP12 exhibits inflammasome properties in some specific infections but acts as a negative regulator in intestinal diseases (81). Recent studies have shown that NLRP12 knockout (NLRP12^-/-^) in a mouse model of antigen-induced arthritis (AIA) with an increased Th17-associated inflammatory response develops more severe arthritis, and NLRP12 negatively regulates STAT3 phosphorylation of the IL-6 pathway (82). Overexpression of NLRP12 inhibited the proliferation of RA-FLSs and downregulated inflammatory cytokines, including IL-6, IL-1β, and TNF-α. NLRP12 knockdown promoted the phosphorylation of NF-κB, ERK, JNK, and p38, indicating NLRP12 is also a negative regulator of inflammation in RA (83).

## Inflammasome in RA

### NLRP3

#### Expression of NLRP3 in Animals

Inflammasomes have been studied in animal models of RA and in humans to help understand their role in pathogenesis. Synovial NLRP3 expression is increased in the collagen-induced arthritis (CIA) model, and positively correlates with radiological destruction and arthritis severity (84, 85). ASC knockout (ASC^-/-^) mice are protected from arthritis, while caspase-1 knockout (caspase-1^-/-^) and NLRP3 knockout (NLRP3^-/-^) mice are susceptible to CIA (86). The expression of NLRP3 inflammasomes is also increased in the adjuvant arthritis (AA) model. Silence of the NLRP3 gene downregulated matrix metalloproteinase (MMP)-1 and IL-1β (87).

#### Expression of NLRP3 in Humans

Several studies have demonstrated that NLRP3 is activated in RA patients. NLRP3 and IL-1β secretion are elevated in peripheral blood mononuclear cells (PBMCs) from RA patients (88, 89). It was also found that miR-33 level was significantly increased in PBMCs from RA patients, which enhanced NLRP3 inflammatory vesicle expression (90). IL-18 and IL-1β levels in bronchoalveolar lavage fluid (BALF) were also elevated in RA-usual interstitial pneumonia (RA-UIP) patients (91). Unlike macrophages or monocytes, NLRP3 mRNA levels, ASC and pro-caspase-1 levels were reduced in neutrophils from RA patients, while the level of active caspase-1 was elevated and positively correlated with the CRP-based 28 joint disease activity score (DAS28-CRP). Caspase-1 activation was not correlated with IL-1β levels but positively correlated with serum IL-18 levels (18). Notably, NLRP3 was activated in CD4^+^T cells of RA patients, and its activation correlated with serum IL-17A concentrations and disease activity. Th17 cell differentiation was inhibited after NLRP3 knockdown, suggesting that NLRP3 not only increased inflammatory cytokines in RA patients but also exerted pathogenic effects by promoting Th17 cell differentiation (92). NLRP3 and ASC expression in synovial tissue of RA patients were higher than those in osteoarthritis patients (93). Interestingly, IL-1β levels were higher in ACPA-positive RA patients, and ACPA may activate the Akt/NF-κB signaling pathway through enhanced interaction with CD147, stimulating IL-1β production by macrophages (94). Two recent studies have shown that calcium-sensitive receptors (CaSR) in RA patients can mediate NLRP3 inflammasome activation, increase IL-1β levels and exacerbate joint and systemic inflammation (95). Complement C1q can act synergistically with PTX3 to promote NLRP3 inflammasome scorching and hyperactivation in RA patients (96).

#### Genetic Polymorphisms of NLRP3 in RA

Genetic polymorphisms of inflammasomes are associated with the inheritance of RA (**Table 1**). It has been shown that single nucleotide polymorphisms (SNPs) in NLRP3 and CARD8 are related to increased susceptibility to RA and response to anti-TNF therapy (97, 98). Carriers of the NLRP3 (rs10754558) gene variant were more likely to have a negative response to anti-TNF treatment (99). A study from Brazil also confirmed that polymorphisms in CARD8 and NLRP3 wererelated to RA susceptibility and disease severity (100). A study from northern Sweden showed that CARD8-X was related to disease severity in early RA (101). Another Swedish study showed that genetic variants in NLRP3 were associated with the risk of transient ischemic attack (TIA) or stroke in RA patients (102). Genetic polymorphisms in cryopyrin (CIAS1) and TUCAN (CARD8) were related to both RA disease severity and susceptibility (104). However, contradictory results were also reported, with polymorphisms in NLRP3 (p.Q705K) and CARD8 (p.C10X) not related to RA susceptibility in French or Tunisian populations (103). While a retrospective study of 1530 patients with RA in Spain concluded that CARD8 rs2043211 gene variants were not associated with the severity of cardiovascular disease development and disease susceptibility in RA patients, it is controversial whether inflammasomes are related to RA-complicated cardiovascular disease (105).

**Table 1:** The relationship between inflammasome SNPs and RA.

	SNP	Study population	Association	Ref.
NLRP3	rs10159239	Caucasian	associated with RA susceptibility and anti-TNF response	(97)
	rs4612666	Denmark	associated with anti-TNF response	(98)
	rs10754558	Denmark	associated with anti-TNF response	(99)
	rs10754558	Brazil	associated with RA susceptibility and severity	(100)
	rs35829419	Sweden	not associated with an increased susceptibility	(101)
	rs35829419	Sweden	associated with an increased risk of stroke/transient ischemic attack	(102)
	rs35829419	France, Tunisia	not associated with an increased susceptibility	(103)
CARD8	rs16981845	Caucasian	associated with RA susceptibility and anti-TNF response	(97)
	rs2043211	Brazil	associated with RA susceptibility and severity	(100)
	rs2043211	Sweden	associated with a worse disease course in early RA	(101)
	rs2043211	Sweden	not associated with any type of CV event	(102)
	rs2043211	Sweden	associated with RA susceptibility and severity	(104)
	rs2043211	France, Tunisia	not associated with an increased susceptibility	(103)
	rs2043211	Spain	not associated with RA susceptibility and the development of CV disease	(105)
NLRP1	rs878329G	Han Chinese	Increase risk of RA	(106)
	rs6502867 T/C	Chinese Singaporean	not associated with risk of RA	(107)
	rs6502867 C/T	Chinese	not associated with risk of RA	(108)
	rs878329 C/G			

SNP, single nucleotide polymorphisms; CV, cardiovascular.

#### Inhibition of NLRP3-Associated Signaling Pathway

Inhibition of NLRP3-associated signaling pathways may become an effective way to treat NLRP3-mediated diseases. Some studies have shown that overexpression of miRNA-20a resulted in reduced NLRP3 expression and decreased secretion of inflammatory cytokines, including MMP-1 and IL-1β. MicroRNA-20a may downregulate Thioredoxin-interacting protein (TXNIP) expression, thereby inhibiting the NLRP3 inflammasome (87). Protectin DX (PDX) was also shown to inhibit NLRP3 expression *via* the miRNA-20a pathway, regulate Treg/Th17 cell homeostasis and significantly delay disease progression in CIA models (109). A recent study showed that IL-6 could induce activation of the NLRP3 inflammasome *via* the cathepsin B (CTSB)/S100A9-mediated pathway and promote joint inflammation in CIA mice, suggesting that the IL-6/NLRP3 pathway may also be a novel target for RA therapy (110). In addition, tofacitinib restores the cellular balance of γδ-Treg/γδ-T17 cells in the CIA model, and a balanced γδ-Treg/γδ-T17 cell ratio inhibits NLRP3 expression and reduces IL-1β secretion (111). Recent studies have shown increased expression of long noncoding RNA myocardial infarction-associated transcript (lncRNA MIAT) in the myocardial tissue and synovium of CIA mice. LncRNA MIAT suppresses TNF-α and IL-1β expression but is inhibited by the ATP-activated NLRP3 inflammasome. Macrophage infiltration is increased in CIA tissues, and LPS-induced macrophage inflammation can in turn upregulate lncRNA MIAT expression; thus, lncRNA MIAT in macrophages may become a new target for RA therapy (112).

### AIM2

With the in-depth study of NLRP3, NLRP3-associated AIM2 inflammasomes as cytoplasmic receptor are becoming the focus of recent research in RA pathogenesis. Pannus is formed in the RA joint due to vascular hyperplasia, and there is a hypoxic microenvironment inside the joint. Hypoxia causes mitochondrial or nuclear DNA damage, and since mtDNA is closer to the respiratory chain, it is more likely to be damaged in oxidative stress. RA patients have higher mtDNA levels in the plasma and synovial tissue than those in healthy controls, and are more likely to activate AIM2 inflammasomes (113–115). A meta-analysis showed that AIM2 gene expression levels were significantly upregulated in PBMCs from RA patients (116). Arthritis-prone mice with AIM2 deficiency exhibited significantly attenuated joint inflammation and histopathological changes (117, 118). Two new studies showed different results: monocytes in RA patients were more likely to release IL-1β in the absence of AIM2 inflammasome signaling (119). The serum AIM2 levels were lower in RA patients than that in healthy controls, while the levels of caspase-1, ASC, IL-1β, and molecules associated with AIM2 inflammasomes, were higher than those in healthy controls, plus positively correlated with the levels of CRP and ESR. AIM2 levels were higher in FLSs of RA patients than those in osteoarthritis (OA), and FLS proliferation was inhibited by silencing AIM2 in FLSs. Therefore, despite the inconsistent results, AIM2 remains a target for RA therapy (120). TRIM11 is a negative regulator of AIM2, which can degrade AIM2 in a p62-dependent manner (121).

### NLRP1

In the adjuvant arthritis (AA) rat model, the NALP1 inflammasome was activated, and carboxyamidotriazole (CAI) reduced proinflammatory cytokine secretion by inhibiting the NF-κB signaling pathway and suppressing NALP1 activation, which may be beneficial for RA treatment (122). An inhibitor of 11 β-hydroxysteroid dehydrogenase 1 (11β-HSD1), bvt2733, has been shown to reduce joint symptoms and decrease serum IL-1β, IL-17, TNF-α and IL-6 levels in CIA mice by inhibiting NLRP1 inflammasomes and NF-κB signaling pathways (123). The purinergic receptor P2X4 antisense oligonucleotide (AS-ODN) plays a therapeutic role in reducing the clinical scores of CIA by inhibiting the NLRP1 signaling pathway (124). A study with a large sample size showed that the NLRP1 and NLRP3 genes were associated with RA, *via* analyzing PBMCs expression profiles in RA patients (125). A study from France also suggested that mutations in the NLRP1 gene may be related to the development of RA (126). A study by Sui et al. showed that the rs878329 G allele in NLRP1 correlated with the risk of RA, and the polymorphism of the NLRP1 gene was associated with the incidence of RA in the Han population (106). However, there were also different results: polymorphisms in NLRP1 rs6502867 T/C were shown not related to the risk of developing RA in Chinese Singaporeans (107), and another study also showed that genetic polymorphisms in NLRP1 rs6502867 C/T and rs878329 C/G were not associated with RA (108). Ethnicities, geographic locations, lifestyles, and the sample sizes may affect the conclusions. Whether NLRP1 diversity is associated with RA susceptibility or severity remains to be investigated in more depth.

### NAIP-NLRC4

Although much less research has been done on NLRC4 in RA, a recent study showed significantly elevated NLRC4 and NLRP3 expression in monocytes from RA patients, supporting a role of inflammasomes in RA (100).

## Inflammasomes and RA Therapy

The role of various inflammasomes is increasingly recognized in autoimmune diseases. Thus, targeting inflammasomesor their associated cytokines may become new strategies for therapeutic intervention. Several inhibitors of inflammasomes have been identified, including those that directly inhibit NLRP3 inflammasomes and indirectly inhibit caspase-1 or IL-1 signaling pathways **(** **Figure 2** **)**.

### NLRP3 Inhibitors

The drug glyburide for the treatment of type 2 diabetes (T2D) selectively inhibits NLRP3 inflammasomes, and the inhibition of NLRP3 by glyburide demonstrates for the first time that selective pharmacological inhibition is feasible (127). Another NLRP3 inhibitor, MCC950, has the same high specificity as glyburide but has no inhibitory effect on AIM2, Pyrin, NLRP1, and NAIP-NLRC4 inflammasomes. It prevents ASC oligomerization by inhibiting NLRP3 activation, downregulating IL-1β secretion (128). A recent study showed that MCC950 could directly target the NACHT domain of NLRP3 and block ATP hydrolysis to inhibit NLRP3 activation (129). Although MCC950 is a potent and specific small molecule inhibitor of NLRP3 and has shown beneficial effects in models of myocardial infarction, atherosclerosis, colitis, airway and skin inflammation, phase II clinical trials of MCC950 in RA were discontinued due to its hepatoxicity (130, 131).

The anti-allergy drug tranilast (TR) is also a direct NLRP3 inhibitor, which binds to the NACHT domain of NLRP3, inhibiting NLRP3 assembly by blocking its oligomerization (132). TR has shown beneficial effects in mouse models of T2D, cryopyrin-associated periodic syndrome (CAPS) and gouty arthritis (132). TR was evaluated for safety and efficacy in CAPS patients in a phase 2 open-label clinical trial (NCT03923140) (131, 133). Bay 11–7082 and parthenolide directly inhibit NLRP3 and also inhibit caspase-1 activity, but are not suitable for clinical development due to the potential for widespread immunosuppression (134). CY-09, oridonin, and derivatives of acrylamide (e.g., INF58) all directly inhibit NLRP3 (135–137). Studies have also shown that human umbilical cord blood-derived mesenchymal stem cells (hUCB-MSCs) ameliorated CIA in the mouse model to a similar extent as etanercept. hUCB-MSCs can modulate multiple cytokine pathways and may be a favorable candidate for the treatment of patients with refractory RA (138). Taraxerol significantly inhibited IL-1β-induced proinflammatory cytokines, including IL-6, IL-8 and TNF-α *in vitro* and inhibited NLRP3 inflammasome expression in a model (139). Cinnamaldehyde (CA) is also a promising drug for RA therapy. It reduces the joint inflammatory response in RA rat models, especially cytokines associated with IL-1β. CA may inhibit the activation of the NLRP3 inflammasome and suppress disease progression by regulating the succinate/HIF-1α axis (140). Recently, RRx-001, a well-tolerated anticancer agent, has been identified as a potent covalent NLRP3 inhibitor and may serve as a new potential therapeutic agent for NLRP3-driven diseases (141). All of the above are in the experimental stage and have not yet been applied for the treatment of autoimmune diseases in humans. Research on NLRP3 inhibitors is rapidly advancing, and the promising compounds with good safety profiles, high specificity and low cost will provide benefits for the treatment of patients.

### Caspase-1 Inhibitors

Caspase-1 is common to all canonical inflammasomes, and the development of selective inhibitors of caspase-1 protease is a hotspot in the pharmaceutical industry in recent years (142). VX-740 (pralnacasan) and its analog VX-765 (belnacasan) can be metabolized to VRT-18858 and VRT-043198, respectively. VX-740 attenuates both RA and OA knee osteoarthritis injury, and VX-765 inhibits cytokine secretion and reduces disease severity in models of skin inflammation as well as RA (143, 144). Although VX-740 showed good anti-inflammatory performance in phase I and II clinical trials in RA patients, the trials were discontinued due to its hepatotoxicity (145). VX-765 failed to meet the stated endpoints in phase II clinical trials, although it reduced the release of IL-1β and IL-18 in mice and reduced seizures in a mouse model of chronic epilepsy (NCT01501383) (143, 146, 147).

### IL-1/IL-18 Blockades

IL-1β and IL-18 are the major inflammatory cytokines activated by various types of inflammasomes and are involved in the pathogenesis of several autoimmune diseases. Therefore, blocking IL-1 and IL-18 would be a more desirable therapeutic strategy. Three biological anti-IL-1 agents have been approved for clinical use: anakinra, a human recombinant interleukin-1 receptor antagonist (IL-1Ra) that competitively inhibits IL-1α and IL-1β; canakinumab, a fully human anti-IL-1β monoclonal antibody; and rilonacept, an IL-1 inhibitor (IL-1 Trap). Anakinra was firstly developed for use in patients who had no response to conventional therapy for RA. Anakinra inhibited disease activity in RA patients but was later found to be less effective than TNF-α blockers (148, 149). Therefore, anakinra is currently used in the treatment of adult Still’s disease (AOSD), Schnitzler syndrome (SchS), and systemic juvenile idiopathic arthritis (SJIA) and has also shown better results and safety in patients with gout (148). Anakinra was found in a recent small multicenter and randomized clinical trial to improve inflammatory and glycemic parameters in patients with RA and T2D (NCT02236481) (150). Since anakinra has a half-life of only 4~6 hours, it requires frequent injections, with the resulting potential risk of infection, whereas canakinumab has a half-life of 26 days and showed a better treatment response and a higher safety profile in patients with active RA in a phase II multicenter randomized and double-blind trial (NCT00784628) (151). Additionally, canakinumab has shown beneficial effects in active SJIA, autoinflammatory recurrent fever syndromes, atherosclerosis, and lung cancer (152–156). The third agent, Rilonacept, is mainly used to treat gout in children and adults with CAPS as well as SchS (157–159).

Currently, two IL-18 blockers are being explored in clinical trials. Tadekinigalfa, a recombinant human IL-18 binding protein, is both effective and safe in phase II clinical trials in AOSD (NCT02398435) (160). GSK1070806 is a recombinant human IL-18 neutralizing antibody currently under phase II clinical trials for the treatment of moderate to severe Crohn’s disease (NCT03681067) (131). If these clinical trials are proved effective, it will be possible to extend inhibitors of IL-18 to the treatment of other autoimmune diseases with abnormally high level of IL-18.

## Conclusions

RA is a complex autoimmune disease caused by multiple environmental and genetic factors. Over the past decades, there have been significant advances in understanding the pathogenesis of RA. Compelling evidence indicates that inflammasomes play a critical role in the RA disease process. In recent years, reports on elucidation of different mechanisms of inflammasome activation and regulation have also made it possible to design effective inflammasome inhibitors. Advanced technologies such as solution-state NMR, X-ray crystallography, and cryo-EM have all contributed to the characterization of the high-resolution structure of receptor/ligand-driven induced conformational changes. Thus, further understanding of the effector mechanisms of inflammasome activation and immune regulation will not only provide new insight in RA pathogenesis but also facilitate the development of novel therapeutic strategies for the treatment of RA and other autoimmune diseases.

## Author Contributions

QJ and DC drafted the manuscript and designed the figures and tables. XW, EH, QW, CW, GY, LL, and DC revised the manuscript. DC, LL, and GY conceived the topic. All authors contributed to the article and approved the submitted version.

## Funding

This work was supported by the National Natural Science Foundation of China (Grant Nos. 81871709, 82071817), Funding for Chongqing International Institute for Immunology (2020YJC10), Hong Kong Research Grants Council General Research Fund (17113319) and Theme-Based Research Scheme (T12-703/19R), Basic Public Welfare Research Plan of Zhejiang Province (LGD21H100001).

## Conflict of Interest

The authors declare that the research was conducted in the absence of any commercial or financial relationships that could be construed as a potential conflict of interest.

## Publisher’s Note

All claims expressed in this article are solely those of the authors and do not necessarily represent those of their affiliated organizations, or those of the publisher, the editors and the reviewers. Any product that may be evaluated in this article, or claim that may be made by its manufacturer, is not guaranteed or endorsed by the publisher.

## Glossary

HLA: human leukocyte antigen
ACPA: anti-citrullinated protein antibody
CCP: cyclic citrullinated peptide
RF: rheumatoid factor
ESR: erythrocyte sedimentation rate
CRP: C-reactive protein
TNF: tumor necrosis factor
PAMPs: pathogen-associated molecular patterns
DAMPs: damage-associated molecular patterns
HAMPs: homeostasis-altering molecular processes
PRRs: pattern recognition receptors
TLRs: Toll-like receptors
NLRs: NOD-like receptors
CLRs: c-type lectin receptors
RLRs: retinoic acid-inducible gene (RIG)-I-like receptors
AIM2: absent in melanoma 2
CARD: caspase recruitment domain
ASC: apoptosis-associated speck-like protein containing a CARD
PYD: pyrin domain
BIR: baculovirus inhibitor of apoptosis protein repeat
NBD: the central nucleotide-binding domain
LRR: leucine-rich repeat
IFI16: human interferon (IFN)-g-inducible protein 16
ATP: adenosine triphosphate
MSU: uric acid crystals monosodium urate
LPS: lipopolysaccharides
NF-κB: nuclear factor-κB
dsDNA: double-stranded DNA
T3SS: type three secretion system
FLS: fibroblast-like synovial
AIA: antigen-induced arthritis
CIA: collagen-induced arthritis
AA: adjuvant arthritis
PBMC: peripheral blood mononuclear cell
SNP: single nucleotide polymorphism
T2D: type 2 diabetes
CAPS: cryopyrin-associated periodic syndrome
